# Moses Atwood Hopkins

**Published:** 1897-04

**Authors:** B. Merrill Hopkinson

**Affiliations:** Baltimore, Md.


### Obituary.

### MOSES ATWOOD HOPKINSON, D. D. S.

Dr. Hopkinson, who died upon January 20th, 1897, after a very brief illness, was born in East Bradford near the Merrimac River in Essex Co., Massachusetts, July 24, 1824. His mother, Maria Atwood Hopkinson, an accomplished lady, died during his infancy. His father, William Hopkinson, an architect, contractor and builder, was at one time engaged in the tobacco business, and was for many years town clerk and town treasurer, and his ancestors for many generations, lived, died, and were buried in that town.

They were of English descent and were related to Francis Hopkinson signer of the Declaration of Independence.

His grandfather married Hannah Balch, a daughter of the first minister of Bradford.

A remarkable trait of the Hopkinson family has always been a love of personal independence. The town of Bradford was noted for its academies. The principal of Bradford academy was Benjamin Greenleaf, the celebrated mathematician. Merrimac Academy had students from the remotest parts of the country.

After receiving an education at this school, the subject of this sketch, at the age of eighteen, secured the position of principal of one of the public schools of Amesbury, Mass. This school had previously been taught by the poet Whittier.

Young Hopkinson subsequently held the position of principal of a successful private school in the adjoining town of Haverhill, Mass. The confinement incident to this employment was unsuited to his taste and habits, and as it was his early intention to become a medical practitioner; he adopted teaching as a temporary employment. He therefore abandoned it, and at the last moment of delay before entering upon professional studies, chose dentistry as his future vocation. After an extended and expensive course of private tuition in Boston, he removed to Baltimore in 1847, that city containing at that time the only Dental College in the United States, where he graduated in 1849. He immediately entered upon a successful practice in Baltimore. Upon the arrival of what he termed the era of "cheap dentistry" he prepared to retire from practice, and of late years was well known in business circles.

Dr. Hopkinson had been twice married, once at the residence of the Roman Catholic Archbishop of Baltimore, and once at the church of Epiphany in Washington, D. C. His first wife whom he married in 1853 and by whom he had a son, B. Merrill R. and a daughter Emily J., was Miss Lizzie Frailey, a sister of Commodore James Frailey, of the U. S. Navy. His second wife died in 1874.

He was a member of the Grand Lodge of Md. Knights of Pythias, a member of the Independent Order of Odd Fellows, and the Protestant Episcopal Brotherhood of Baltimore.

At an early period in his life Dr. Hopkinson became a member of the Unitarian church, but in 1850 he entered the Roman Catholic Church, uniting with the Cathedral congregation of Baltimore, and it was there that both his children were baptized. In 1860 he united with Grace Episcopal Church of Baltimore and during the last twelve or fifteen years of his life he was a member of old St. Pauls.

He was a genial, warm hearted, generous man; never thinking of himself, but lavishing his bounteous store of love and affection upon those dear to him, and never regarding anything a labor or trouble that could lighten the burdens of those he loved, or give them a moment of happiness. His children, for whom he lived, are grateful to the Almighty God for the blessed inheritence of his devoted life, and can only pray that they may so live that they will receive his blessing from his place of rest.

Having devoted his life to the investigation of religious subjects, and to the search for a system adapted to the restoration of harmony to the church, and having been baptized by an Unitarian Minister, and by a Roman Catholic Priest, and having been confirmed by a Roman Catholic Archbishop and a Protestant Episcopal Bishop, it may be interesting to state the conclusions to which he arrived, and I quote his own words:

"That all religion's are necessary." "The Christian religion is of infinite importance to mankind." "The only way harmony can be restored to the church is for each denomination to abjure whatever is anti-christian in its tenets, doctrines or practices; in other words to teach christianity pure and simple, and not denominationalism." Intrinsically no one of the various sects into which the true christian church is divided is any better than another, and it is the height of presumption to make the claim. In the judgment of the individual, one may be better suited than another to his state of development, as therefore, a choice by an individual of one instead of another on account of its intrinsic superiority cannot be made—one being as good as another for the purposes for which the church was instituted. A selection must be made from other considerations, chiefly because it is necessary to unite with one of those in order to become a member of the church of Christ."

I know my dear Father to have been a consistent christian, and a paper found in his pocket at the time of his death, written by him and easily recognized, reads, "the principal business of this life is to serve God."

Sorrow and gloom is all pervading, as his bright smile, cheery words, and manly grip are no more; I have lost a Father, a guide, a counsellor, a companion, and, a friend; but in the future appears the bright promise that we shall meet again, and when I shall enter the shadows of the great unknown, I shall there be greeted by one who always loved me, and who is the best friend I have ever had, my own dear father.

B. MERRILL HOPKINSON, D. D. S., M. D.
Baltimore, Md.

